# Into the wild: how living labs are being used to connect people with dementia to nature—a mini review

**DOI:** 10.3389/frdem.2026.1822803

**Published:** 2026-06-23

**Authors:** Cassandra J. Thomson, Sharon Stoddart, Hoang Nguyen, Shuzhen Joanna Sun, Alice Rota-Bartelink, Pauline Marsh

**Affiliations:** 1 Venture Out Living Lab and Research Group, Wicking Dementia Research and Education Centre, University of Tasmania, Hobart, TAS, Australia; 2 Australian Institute of Health Innovation,Macquarie University, Sydney, NSW, Australia

**Keywords:** dementia, environmental design, green space, living lab, long-term care, nature, participatory design, quality of life

## Abstract

With the global rise in dementia diagnoses and no available cure, there is an urgent need for accessible, enjoyable, and holistic interventions that support people experiencing dementia to live well. Nature engagement has been shown to offer wide-ranging benefits, including improved mental health, physical activity, cognitive stimulation, and social connection, but many people with dementia face barriers to accessing natural environments due to mobility challenges, cognitive changes, poor environmental design, and dementia-related stigma. Innovative approaches are needed to make nature more accessible and meaningful for this population. One promising solution is the use of living labs: real-world, user-centred environments where researchers, service providers, people with dementia, and care partners can co-create and test solutions. Living labs are grounded in principles of collaboration, inclusivity, and continuous feedback, and they offer a dynamic space for innovation tailored to the living experiences of those involved. Living labs have been applied in dementia care research, although much of this work has focused on indoor settings, technological tools, and aged care systems. How living labs have been used to help people living with dementia access the outdoors and connect with nature is an underexplored area, but one which holds exciting potential to improve the lives of people with dementia. This mini review summarises the available academic literature examining the nexus between living labs, nature, and dementia and offers suggestions for future work in this area.

## 1 Introduction

With advances in early detection and diagnosis of dementia, and in the absence of a cure, there is an ethical imperative to provide accessible, enjoyable, and holistic interventions that improve the quality of life and wellbeing of people experiencing dementia. Contact with nature is recognised as having wide-reaching health and wellbeing benefits for humans, and this is particularly true for people living with dementia (Bennett et al., 2022; Mmako et al., 2020). Engagement with green spaces and nature-based activities can promote physical activity, provide fresh air and daylight (Clark et al., 2013; Nguyen et al., 2025), and in turn support appetite and sleep, which are often affected by disease progression. Cognitive benefits include improved memory, attention, orientation and awareness of surrounds, alongside multisensory stimulation. Natural environments can also facilitate communication (improved verbal expression, reminiscence) and social interaction for people living with dementia, which contributes to social connectedness and a sense of belonging (Clark et al., 2013; Nguyen et al., 2025). Mental health benefits such as increased positive emotional states (joy, pleasure, comfort) and decreased negative emotional states (anxiety, stress, agitation) are accompanied by opportunities to exercise independence, control, and autonomy through positive risk-taking (Bennett et al., 2022; Mmako et al., 2020). Beyond the physical and mental health benefits, it is evident that engagement with nature offers people living with dementia meaningful, enriching, and restorative experiences.

Despite these widely recognised benefits and the deep appreciation that many people with dementia have for nature, time spent outdoors and in natural environments often declines; retreating indoors starts in the early stages when people typically live at home (Duggan et al., 2008), and intensifies in the later stages when a transition to long-term care can occur (van den Berg et al., 2020). Symptoms associated with dementia (e.g., memory loss, disorientation, confusion, mobility decline) can reduce confidence and willingness to access outdoor green spaces and worries about falling, incontinence, and getting lost are common (Clark et al., 2013; Duggan et al., 2008). Risk aversion is prominent amongst people living with dementia, care partners, and service providers. Beyond this there are barriers to accessing nature that can be categorised as social (e.g., dementia stigma in the community and limited dementia awareness amongst green space providers), environmental (e.g., poor accessibility and dementia-inclusive design), and logistical (e.g., due to staff resourcing, transport, carer costs, or lack of nearby nature) (Mapes et al., 2016). Solutions are needed to address these barriers to ensure that people with dementia are not excluded.

In recent decades, living labs have emerged as a promising methodological approach to address the range of complex problems associated with dementia that affect individuals, their families, and whole societies. While various definitions of a living lab exist, a leading definition comes from the European Network of Living Labs who describe a living lab as a “user-centred, open innovation ecosystems based on a systematic user co-creation approach, integrating research and innovation processes in real-life communities and settings” (European Network of Living Labs, 2026, para. 2). Living labs occur across diverse settings including simulated home environments, actual home environments, public institutions such as universities and schools, and entire cities and regions (i.e., Urban Living Labs). To-date, living lab research in dementia has primarily focused on indoor spaces, activities, and technologies (Figueiredo et al., 2024; Verloo et al., 2021). This research often comes from the field of human-computer interaction where embodied interactions with technology are meaningfully observed in real-life environments. Ambient assisted living (i.e., the unobtrusive use of technology such as sensors and smart technology to support safety and daily living) is a common approach. The living lab research involving indoors spaces and technologies has diverse aims such as improving the person living with dementia’s quality of life and cognition, promoting their independence and autonomy, and ensuring their safety and reducing care partner stress (Figueiredo et al., 2024).

The phrase ‘research in the wild’ is often applied to living labs due to their setting in real-life environments. Here, we examine the application of living labs in the ‘wild’ in its truest sense of the word (i.e., in outdoor natural environments). In particular, we explore how living labs have sought to address the nature disconnection challenge that many people living with dementia face (Barrett et al., 2019; Nguyen et al., 2025). This mini review summarises the current literature, identifies gaps, and discusses opportunities for the development of living labs seeking to connect people with dementia to nature, thereby improving quality of life and wellbeing.

## 2 Methods

The mini review was guided by the research question “have nature-based or outdoor interventions been integrated with a living lab approach in dementia research? If so, how? And what did they find?” The Population/Concept/Context (PCC) framework was applied, with studies involving the following sought: people living with dementia (population), living labs (concept), and outdoor access, natural environments, and/or participation in outdoor, nature-based activities (context). Database searches of MEDLINE Complete, PsycINFO, Embase, PubMed, CINAHL, Scopus, Web of Science, JSTOR, Environment Complete, Informit, GreenFILE were conducted. Search terms included “living lab*,” dementia OR “mild cognitive impairment” OR “neurocognitive disorder” OR alzheimer* OR Parkinson* OR “Lewy body,” and natur* OR outdoor* OR green* OR wild* OR environment* OR horticultur* OR garden* OR park* OR farm* OR agri* OR rural OR blue OR forest OR eco. Peer-reviewed and grey literature published in English until December 2025 were searched. Although the emphasis for the mini review was on recent literature, no strict date limits were applied in order for foundational literature that provided important context for developments in this emerging area to be considered by the authors. All results were screened by two authors (CT, SS). Studies associated with four ageing and dementia-focused living labs were identified. In cases where a study’s association with a living lab was unclear, authors were emailed for clarification. Relevant studies associated with these living labs fit into three broad categories: green care farms, green spaces within built environments, and nature-integrated technology.

## 3 The Limburg living lab in ageing and long-term care, and green care farms

The Limburg Living Lab in Ageing and Long-Term Care, based in the Netherlands, is a well-established example of a living lab dedicated to improving the experience of ageing and quality of long-term care. Established in 1998, it has created a sustainable model for scientific research and practice innovation (Verbeek et al., 2023). Rather than a physical space, the Limburg Living Lab is a network that brings together researchers, older adults and families, professional caregivers, managers, policymakers, and educators (Verbeek et al., 2020). Its core partnership between two universities and nine long-term care providers spans roughly 185 care sites, 27,000 staff, 50,000 clients, and a large cohort of researchers and PhD students (Verbeek et al., 2023). A distinctive feature is its system of joint appointments, known as ‘Linking Pins’, in which scientific researchers are embedded in aged care settings, and practice-based personnel are embedded in research settings. This reciprocal structure strengthens interdisciplinary collaboration and reflects a shared commitment from the Living Lab partners to improving the quality of research, education, and practice in ageing and long-term care.

One key focus area of the Limburg Living Lab is Green Care Farms (GCFs). GCFs are an innovative approach to care that integrates agricultural activities with care for diverse client groups, including people living with dementia (de Bruin et al., 2020). GCFs offer adult day services or long-term care within health-promoting environments with outdoor spaces, animals, plants, and a familiar home-like setting (de Bruin et al., 2020). These environments provide rich sensory stimulation and enable engagement in a range of indoor and outdoor activities such as meal preparation, walking, gardening, feeding and viewing animals, collecting eggs, and woodworking (de Boer, 2017; Rosteius et al., 2025). Meaningfully embedded in everyday farm life, such activities can foster feelings of commitment and responsibility and allow individuals to contribute to their environment while receiving care (Buist et al., 2018). By combining an enabling physical environment with a care philosophy emphasizing individual capabilities and strengths (de Bruin et al., 2017), GCFs aim to promote purposeful occupation and social participation. Research on GCFs providing dementia care in the Netherlands, one of the forerunners in this innovative form of care provision, has largely been conducted within the Limburg Living Lab network. Although similar GCFs models exist internationally (e.g., Anderson et al., 2017; Yamazaki et al., 2019), the Dutch model is unique in its integration with a living lab.

Partnerships between the Limburg Living Lab and GCFs supporting people living with dementia have generated a substantial evidence base. Published studies show diversity in design, varying in purpose (exploratory, descriptive), time frame (cross-sectional, longitudinal), methodological tradition (ethnography, phenomenology, case study), research approach (qualitative, quantitative, mixed methods), and data collection methods (observations, interviews, informal conversations, focus groups, questionnaires, and archival data). Environmental assessments indicate that GCFs score highly across domains associated with a positive physical environment for people living with dementia, particularly in relation to privacy and autonomy, view and nature, orientation and routing, and domesticity (de Boer et al., 2018). Research also indicates that GCFs provide sensory-rich environments and support engagement in purposeful activities, shared responsibilities, and a sense of community (Rosteius et al., 2022a,b). Compared with residents of traditional nursing homes, residents of GCFs demonstrate significantly higher levels of active engagement in domestic and nature-related activities, as well as greater social interaction (de Boer et al., 2017a, Chapter 4 of thesis). Higher quality-of-life scores have also been reported for people living with dementia at GCFs, particularly in the domains of positive affect, social relations, and meaningful occupation (de Boer et al., 2017b, Chapter 6 of thesis). GCFs additionally support reciprocal care relationships between staff and family caregivers (Smit et al., 2022). This may contribute to the overall experience of informal caregivers, who tend to be more positive about the physical environment, available activities, and person-centred care at GCFs than other types of nursing homes (de Boer et al., 2019). Overall, existing evidence suggests that GCFs offer a promising alternative care setting for people living with dementia.

Situating GCFs within an established living lab appears to strengthen both the quality and sustainability of the associated research and care. The living lab infrastructure enables rapid development and testing of new approaches through co-creation with key stakeholders (de Boer et al., 2021). It supports the development of long-term trusting relationships between researchers, care staff, residents, and families (Rosteius et al., 2022a,b; Rosteius, 2025), which is vital for the collection and interpretation of rich and detailed longitudinal data. Moreover, collaboration amongst the network of researchers, care staff, residents and family members creates a supportive environment to successfully introduce a radically different care philosophy (de Bruin et al., 2017) – one that embraces residents’ dignity of risk.

## 4 Green spaces within built environments

In Green Care Farms, residents live within spacious, stimulating natural environments. In contrast, traditional aged care facilities typically offer gardens only within or adjacent to the built environment—if they are provided at all. Current aged care policy increasingly expects long-term care settings to support residents in seeing, accessing, and spending time outdoors, and in maintaining contact with nature (Fleming et al., 2020; Seemann et al., 2024). Miller and Burton (2023) support this approach: they argue in favour of biophilic aged care design that emphasises and facilitates human connection with nature, and for moving away from conventional institutional models. Achieving such outcomes requires collaboration among architects, designers, aged care providers, policymakers, and end users. A living lab can facilitate this interdisciplinary work.

Despite substantial research on gardens and green spaces in care facilities, little of it has been conducted within a living lab context. Although engagement with gardens can help create a sense of home for residents (Tsai et al., 2020), evidence shows that such spaces are often underused, particularly by residents with mobility limitations (van den Berg et al., 2020; Velde-van Buuringen et al., 2025). Contributing factors to underuse include poor accessibility, staff risk-aversion, and limited meaningful activities or points of interest. A living lab approach with genuine co-creation with end-users (residents, families, staff) may help overcome these barriers and ensure outdoors spaces align with users’ needs and preferences.

Few studies report on living lab processes; however, one example published by authors affiliated with the North West Coast Living Lab in Ageing and Dementia and Limburg Living Lab in Ageing and Long-Term Care describes the early coproduction of an outdoor space in an urban care home (Giebel et al., 2022). Interviews and focus groups with staff and family members highlighted the importance of accessibility, sensory features, social spaces, and opportunities for active participation for residents with dementia. Although people with dementia were intended to be included, they ultimately were not due to consent capacity issues. The study reports only the initial stage of coproduction phase, and at the time of writing the final outcomes are unpublished.

The Living Lab Fondation Médéric Alzheimer (Charras et al., 2020) project details collaboration among three French institutions to design dementia-friendly outdoor spaces. The project focused on creating an outdoor space linked to an aged care facility that could also support community and intergenerational use. A conceptual framework, a workshop with landscape architects, and input and feedback from end-users informed the design. The authors acknowledge the difficulty of balancing safety and autonomy in outdoor spaces shared by residents and the broader community. Final outcomes have not been reported, aside from a website description of a horticultural therapy study conducted in the resulting garden (Fondation Médéric Alzheimer, 2026). This leaves important questions unanswered, for example, how effectively did the living lab approach address the complex challenge of balancing safety with inclusive community access? Reliance on internal, secure green spaces may limit residents’ opportunities for social engagement, physical activity, autonomy, and dignity of risk. More research and comprehensive reporting are needed to understand the potential of living labs in shaping outdoor environments in aged care.

## 5 Nature-integrated technology

Although this mini review focuses on how living labs support direct engagement with nature in dementia research, it is also important to acknowledge work on nature-based technologies within living lab environments. Several projects integrate natural elements—such as visual scenes or ambient sounds—into technological systems to evoke some of the psychological and physiological effects associated with time in nature. Relevant examples come from the Design-Driven Living Lab and Expertise Center Dementia and Technology (Brankaert, 2016; Brankaert and Den Ouden, 2017).

One such innovation, Qwiek.up, is a media projection system that displays immersive videos (e.g., forest walks, starry skies, farm visits) accompanied by ambient audio (Brankaert and Den Ouden, 2017). Introduced across three case studies in care homes and day programs in Germany and the Netherlands, the Qwiek.up project demonstrated the value of testing in real-life contexts in uncovering varied preferences, difficulties, and usage patterns. Although it can display personalised content like family slideshows, residents appeared to prefer and respond best to the nature-based modules. Other prototypes include AmbientEcho, an interactive media system featuring a virtual window, photo frame, audio, and lighting display curated for people with mid- to late-stage dementia (Thoolen et al., 2020), and the Vita pillow, a sound device for people with advanced dementia (Houben et al., 2020). Both incorporate nature-based visuals and/or audio, eliciting responses such as reminiscence, conversation, reduced stress, playfulness, and aesthetic enjoyment.

Living labs have also supported technological innovations that can enable more direct engagement with nature and the outdoors. For example, the Design-Driven Living Lab developed the Homing Compass, a simple, non-intimidating device that always points home to help address disorientation and fear of getting lost—key barriers to outdoor mobility for people living with dementia (Brankaert et al., 2020a). Another prototype, the ExperienceSeat (Brankaert et al., 2020b), is a garden in a dementia care facility integrated with an audio system that plays stories and poems to enhance engagement during outdoor visits. While promising, limited evidence is available regarding the effectiveness of these prototypes in practice.

Although nature-based technologies can offer meaningful benefits, they should complement rather than replace opportunities for people with dementia to spend time outdoors. Direct engagement with nature provides unique advantages not achievable through technological substitutes, including sunlight exposure which supports Vitamin D levels (Feehan et al., 2023), regulates circadian rhythms, and helps maintain sleep quality (Düzgün and Akyol, 2017). Other benefits from direct engagement with nature include opportunity for purposeful activity, rich multisensory stimulation, and incidental social connection (Barrett et al., 2019). Failing to provide people living with dementia, particularly those in long-term care with mobility issues, direct access to nature is a human rights issue (Evans et al., 2022). Ethical critiques highlight the risk of over-reliance on technology in dementia care, including concerns expressed by people living with dementia and carers (Alzheimer Europe, 2025). They demand that technologies should align with human rights principles and function as part of a wider ethical and relational ecosystem of care. Accordingly, nature-based technologies should serve as one component within a wider suite of interventions to support wellbeing.

## 6 Discussion—limitations, gaps, and opportunities

The literature in this area has several limitations that reflect broader challenges in living lab research. A key issue is that living lab activities are often underrepresented in traditional academic publications, which generally offer limited space to describe living lab procedures and processes in-depth (Hossain et al., 2019). Aside from PhD theses, which allow detailed reporting, and editorials, which offer more flexibility, conventional peer-reviewed manuscripts tend to be outcome-focused with little scope to explain the living lab’s role (Berberi et al., 2021). This often leads to a disconnect between processes and outcomes, with descriptions of collaboration, co-creation, and iterative cycles separated from research evaluation (Slattery et al., 2020). Although some studies thoroughly document co-creation processes, stakeholder engagement, and iterative development, these same studies provide limited evaluation of outcomes (Kõiv-Vainik and Müller, 2026). Additionally, much living lab work with strong industry ties is reported outside academic channels, such as organisational websites, where information is brief and outcome reporting limited (Leminen and Westerlund, 2019). This creates challenges for identification of relevant living lab work and transparent reporting of outcomes and impact. Finally, the literature reviewed here, and the broader living lab evidence base, is largely dominated by work from Western Europe and Scandinavia, restricting the generalisability of findings (Berberi et al., 2021; Mukherjee et al., 2023); although living labs are gaining traction in other regions.

This mini review identifies three main areas where living labs have explored solutions for connecting people living with dementia to nature: (1) green care farms, (2) green space within built environments, and (3) nature-integrated technology. Although promising, this remains an emerging field, with most work conducted in formal care settings. A significant gap concerns community-based research, despite the fact that most people with dementia live in the community. While existing living lab scholarship has established important foundations, there is a clear need for more community-centred, participatory work. This represents an exciting and largely unexplored direction for future living lab activity. Existing work has centred in and around the home, starting with nature-integrated technology that can be experienced indoors, moving out to green spaces attached to the home, and expanding out further to live-in farms. Extending beyond these settings into the wider community presents an opportunity to co-create and test solutions in more expansive, immersive, and varied natural environments.

From a design and methodological perspective, future living lab work in community green spaces can draw on established approaches from traditional living labs, particularly those focused on technological solutions in indoor contexts. This approach includes moving through phases of exploration, experimentation, and evaluation with people living with dementia as co-creators, and applying common methods such as focus groups, interviews, workshops, surveys, and experiments (European Network of Living Labs, 2025; Figueiredo et al., 2024). However, important divergences are expected in these outdoor and community-based contexts. Participation by people living with dementia may need to be supported through more context-sensitive and embodied approaches, such as walking interviews, *in situ* observation, and sensory engagement (Birt et al., 2023; Chaudhury et al., 2025). While methodological flexibility is a defining strength of living labs, in dementia research it is also essential for enable meaningful involvement, particularly given the potential impacts of the condition on cognitive ability, sensory processing, speech and language, and mobility. Further differences arise in relation to design processes and outcomes. For instance, the rapid prototyping and iterative design common to technological solutions may not be feasible beyond a certain stage with, for example, the design and landscaping of a community green space. Additionally, the potential scalability associated with product-based designs is less likely to apply in these community settings where the outcome or solution is inherently placed-based.

Outside of dementia research, there are relevant examples of living lab projects based in community green spaces, including the Go Slow Trail at Macquarie University (Bosanquet et al., 2024; Llyod, 2021). Designed for university students and staff, this trail featuring sensory installations is a demonstration of a living lab working to enhance community engagement with nature to support wellbeing. Such examples offer useful guidance, but future dementia-focused work must be tailored to the specific needs of people living with dementia, their families, and carers.

Research is responding to a moral imperative to ensure that studies involving people living with dementia improve quality of life and support wellbeing, while upholding human rights. This review suggests that the living lab model, of collaborative in-the-wild, real time, interdisciplinary, iterative research shows great potential to achieve this goal. Nature-based living lab research involving people with dementia in their own communities is still in its infancy. Nonetheless, this is an optimistic time and the convergence of social, health, and environmental research priorities within living lab practice can provide a fertile ground for addressing the complex challenges associated with dementia.
